# Endogenous CRISPR-Cas System-Based Genome Editing and Antimicrobials: Review and Prospects

**DOI:** 10.3389/fmicb.2019.02471

**Published:** 2019-10-25

**Authors:** Yingjun Li, Nan Peng

**Affiliations:** State Key Laboratory of Agricultural Microbiology, College of Life Science and Technology, Huazhong Agricultural University, Wuhan, China

**Keywords:** endogenous CRISPR-Cas system, genome editing, antimicrobials, phage therapy, *Mycobacterium tuberculosis*

## Abstract

CRISPR-Cas systems adapt “memories” *via* spacers from viruses and plasmids to develop adaptive immunity against mobile genetic elements. Mature CRISPR RNAs guide CRISPR-associated nucleases to site-specifically cleave target DNA or RNA, providing an efficient genome engineering tool for organisms of all three kingdoms. Cas9, Cas12, and Cas13 are single proteins with multiple domains that are the most widely used CRISPR nucleases of the Class 2 system. However, these CRISPR endonucleases are large in size, leading to difficulty for manipulation and toxicity for cells. Most archaeal genomes and half of the bacterial genomes encode different types of CRISPR-Cas systems. Therefore, developing endogenous CRISPR-Cas systems-based genome editing will simplify manipulations and increase editing efficiency in prokaryotic cells. Here, we review the current applications and discuss the prospects of using endogenous CRISPR nucleases for genome engineering and CRISPR-based antimicrobials.

## Introduction

Site-specific nucleases which can introduce targeted DNA double-strand break (DSB) are employed for genome editing, facilitating the identification, characterization, and modification of important genetic element in the study of biological processes. Three kinds of designer nucleases have been used for targeted DNA DSB formation: zinc finger nuclease (ZFN), transcription activator-like effector nuclease (TALEN), and clustered regularly interspaced short palindromic repeat-associated (CRISPR-Cas) system. The first two nucleases are protein-driven genome editors (Kim et al., 1996; Moscou and Bogdanove, 2009), while the CRISPR-Cas system is a RNA-guided genome editing system (Barrangou et al., 2007). Currently, CRISPR-Cas9 and CRISPR-Cas12a (formerly called Cpf1) systems from bacteria have been developed as efficient genetic manipulation tools for a wide range of species in three domains of life (Doudna and Charpentier, 2014; Shalem et al., 2014; Zetsche et al., 2017). Although the heterogenous CRISPR-Cas system based-tools such as Cas9 and Cpf1 show high genome editing efficiency, they also have many limitations. For example, the intracellular environment and living conditions may affect the activity of the commonly used CRISPR nucleases in some extremophile organisms. Moreover, either heterologous Cas9 or Cpf1 is difficult to introduce into some bacteria and archaea due to their intrinsic protein toxicity (Zhang et al., 2018). Notably, a better strategy for genome editing in prokaryotes that already carry an active CRISPR-Cas system of DNA cleavage is to utilize their endogenous CRISPR-Cas systems.

Endogenous CRISPR-Cas system-based genetic tools can be used for several purposes: prokaryotic genome engineering, mobile genetic element engineering, as well as the antimicrobials against pathogens and microorganisms carrying antibiotic resistance genes. In this review, we introduce basic concept and principle of CRISPR-Cas systems, and summarize pre-existing applications of the endogenous CRISPR-Cas systems for genome editing in bacteria and archaea. In addition, we will focus on the use of CRISPR-based antimicrobials. Furthermore, the future directions and outlook of endogenous CRISPR-based applications are discussed.

## CRISPR-Cas Systems

**Figure 1:**
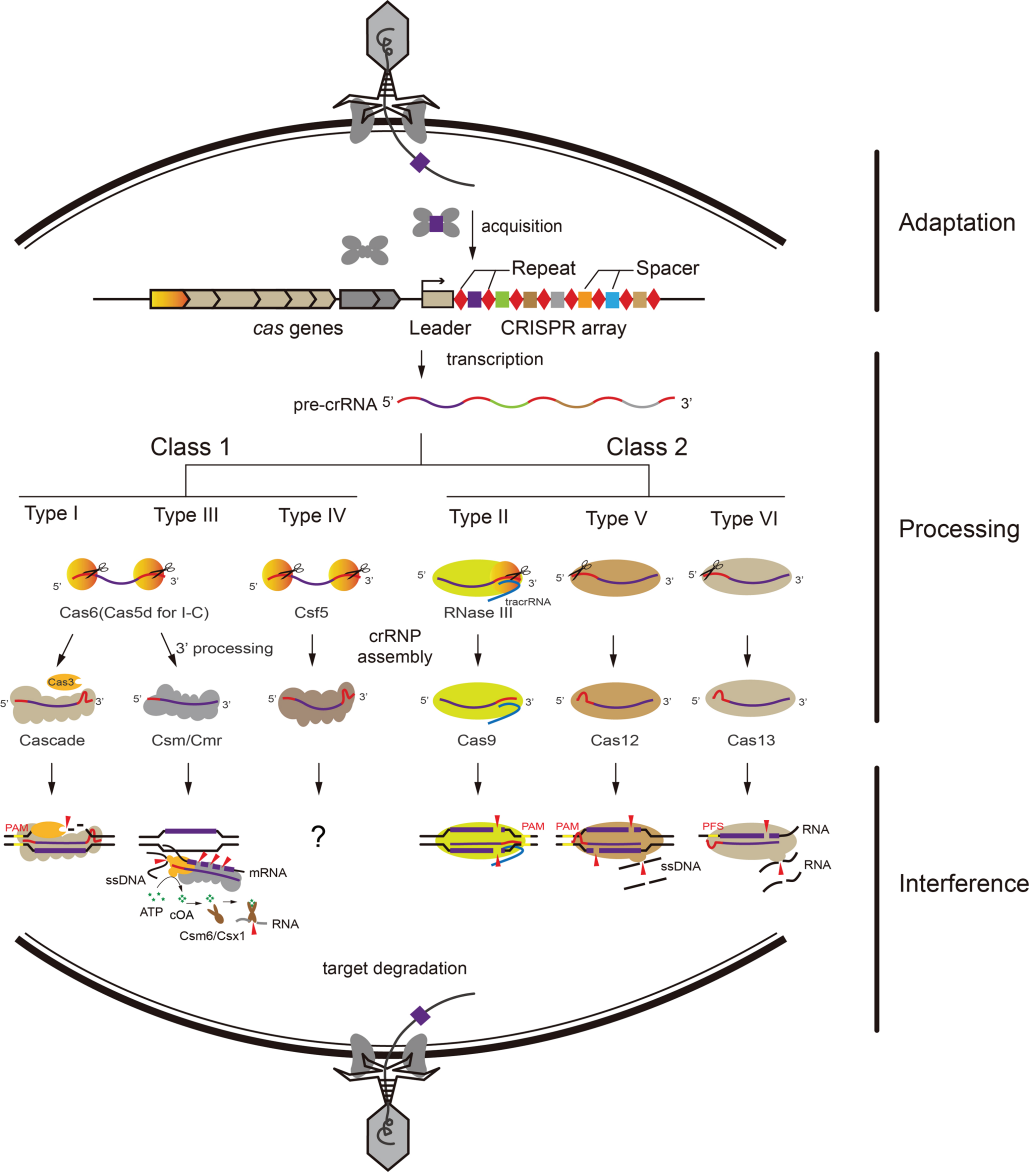
Overview of adaptive immunity by CRISPR-Cas systems. The adaptive immunity by CRISPR-Cas systems functions in three stages: adaptation, processing, and interference. General pathways for Class 1 and Class 2 CRISPR-Cas systems exist. (1) Short fragments of invading nucleic acids are acquired as new spacers into the CRISPR locus by adaptation proteins. (2) The CRISPR locus is transcribed into pre-crRNA. In Class 1 systems, the pre-crRNA is processed into mature crRNAs by a CRISPR-associated nuclease (Cas6 for type I and III, Cas5d for type I-C, Csf5 for type IV). In Class 2 systems, Cas9 binds and stabilizes the tracrRNA:crRNA duplex and further recruits RNase III for crRNA processing. Type V and VI effector proteins process nuclease activity for crRNA maturation by themselves. (3) Mature crRNAs guide CRISPR-Cas effector complexes to cleave invading nucleic acids *via* their respective mechanism, relying on sequence complementarity between the crRNA and the target sequence.

CRISPR-Cas systems have been found in ~45% of bacterial and ~87% of archaeal genomes, which provide an adaptive defense against invading nucleic acids, such as viruses and plasmids (Barrangou et al., 2007; Grissa et al., 2007; van der Oost et al., 2014). This remarkable feature of CRISPR-Cas systems, which are composed of alternating identical repeats and unique spacers, was first recognized in 1987 in *E. coli* K12 (Ishino et al., 1987). Subsequently, three groups found that the spacer sequences in CRISPR arrays match phage genomes and plasmids, which hinted CRISPR-Cas might be a defense system for prokaryotes (Mojica et al., 2005). Adjacent to the CRISPR array, a series of genes encoding the CRISPR associated (Cas) proteins that control the three functional stages of CRISPR adaptive defense: adaptation, processing, and interference. In the best-studied model for adaptation stage, the type I-E CRISPR-Cas system of *E.coli*, two highly conserved nucleases (Cas1 and Cas2) form a stable complex (Nunez et al., 2014) with two Cas1 dimers linked by one Cas2 dimer, that are minimally required for *de novo* spacer acquisition (Yosef et al., 2012). Notably, a dedicated transcriptional regulator is required for activation of adaptation genes for uptake of naive spacers in *Sulfolobus islandicus* REY15A (Liu et al., 2015, 2017b). The second stage is defined as crRNA processing (Bhaya et al., 2011). During the final stage of CRISPR-Cas-meditated immunity, Cas proteins and mature CRISPR RNA (crRNA) form a ribonucleoprotein complex (RNP) that recognizes and cleaves invading foreign DNA (or RNA) *via* base pairing of crRNA and target nucleic acids (Figure 1, see details below) (Brouns et al., 2008).

## Interference by CRISPR-Cas Systems

Based on the differences between the interference complexes, CRISPR-Cas systems are classified into two classes that are subdivided into six types: Type I, III, and IV for Class 1 and Type II, V, and VI for Class 2 (Makarova et al., 2015; Mohanraju et al., 2016). Class 1 systems employ multi-subunits, while in contrast, Class 2 systems employ a single protein and crRNA in RNP complex against invaders (Shmakov et al., 2015). Nearly all characterized CRISPR-Cas systems (except type III) mediate both adaptation and interference to the foreign genetic elements by specifically recognizing a short sequence located at the 5′-flanking region of the protospacers, called the protospacer adjacent motif (PAM) (Peng et al., 2013; Westra et al., 2013; Anders et al., 2014; Li et al., 2014; Wang et al., 2015; Yamano et al., 2017).

Type I system employs a multi-subunit ribonucleoprotein complex termed CRISPR-associated complex for antiviral defense (Cascade) for target DNA recognition and cleavage (Brouns et al., 2008). Cas6 family endoribonucleases recognize and process the CRISPR repeat RNA in almost all type I systems (Cas5d for subtype I-C) (Carte et al., 2008; Garside et al., 2012; Sefcikova et al., 2017). Cascade first recognizes the PAM sequence on the target DNA, and triggers the recruitment of the endonuclease Cas3, which initiates degradation of the non-target strand (Mulepati et al., 2014; Hayes et al., 2016).

Similar to type I complexes, type III systems employ crRNA-bound multi protein complexes for antiviral defense and target both DNA and RNA substrates. The target RNA capture was mediated by a seed motif at the 3′-end of crRNA and the rear-end subunit Cmr1 (Li et al., 2017; Pan et al., 2019). Following the backbone-mediated target transcript cleavage with 6-nucleotide spacing, a RNA-activated DNA cleavage is carried out by the HD (Histidine-aspartate) nuclease domain of the Cas10 subunit (Goldberg et al., 2014; Samai et al., 2015; Elmore et al., 2016; Estrella et al., 2016; Kazlauskiene et al., 2016; Han et al., 2017; Liu et al., 2017c). Meanwhile, the cyclic oligonucleotide (cOA) synthetase activity is catalyzed by the Palm domain of Cas10 which is also activated by the cognate target RNA, and the cOA signaling pathway coordinates the type III RNP complexes and CARF (CRISPR-associated Rossmann fold) domain ribonucleases (Csm6/Csx1 families) to prevent viral infection and propagation (Kazlauskiene et al., 2017; Niewoehner et al., 2017; Han et al., 2018; Wang et al., 2019; You et al., 2019). Type IV systems are also included among the Class 1 CRISPR-Cas systems and harbor multiple subunits, but lack apparent adaptation modules (*cas1* and *cas2*) and interference nucleases (*cas3* or *cas10*) (Koonin et al., 2017). However, the activities of type IV systems are not clear, except the limited knowledge about crRNA maturation and RNP formation (Ozcan et al., 2018).

Type II, V, and VI CRISPR-Cas systems only employing a single protein and crRNA in the RNP complex against invaders are included in Class 2 systems. For CRISPR RNA maturation, Cas12a of type V and Cas13 of type VI can process crRNA themselves (Abudayyeh et al., 2016; Dong et al., 2016; East-Seletsky et al., 2016; Fonfara et al., 2016); however, Cas9 of type II processes the crRNA with help of tracrRNA and RNase III (Deltcheva et al., 2011). Unlike Cas9 of type II that generates a blunt double-strand break, which utilize diverse PAMs on the non-target strand (Fonfara et al., 2014; Nishimasu et al., 2014), Cas12a and Cas12b cleave target DNA *via* a staggered DNA double-stranded break (Figure 1), and recognize the T-rich PAM on both DNA strands (Fonfara et al., 2016; Gao et al., 2016; Yang et al., 2016). Type VI systems encode the single effector protein Cas13 that cleaves target ssRNA, and the target RNA complementary to the crRNA can activate Cas13 to degrade collateral ssRNAs, similar to the ssDNA cleavage activity of Cas10 in type III systems (Shmakov et al., 2015; Abudayyeh et al., 2016; East-Seletsky et al., 2016; Smargon et al., 2017; Liu et al., 2017a); this property of Cas13a has been used for pathogen detection (East-Seletsky et al., 2016; Khambhati et al., 2019). Moreover, Cas12a and Cas12b RNP complexes can also catalyze non-specific ssDNA cleavage when bound with a complementary target ssDNA as an activator (Figure 1; Chen et al., 2018; Li et al., 2018).

## Harnessing Endogenous CRISPR-Cas Systems for Genome Editing

Genome editing is an efficient technology for facilitating the identification and characterization of genes in biological studies. Early versions of genome editing techniques include homing endonucleases (HEs), ZFNs, and TALENs. Homing endonucleases are sequence-specific enzymes that recognize and cleave DNA, which span 12–40 bp at the target sites. By making site-specific double-strand breaks (DSBs) in the target genes, these nucleases can stimulate DNA repair mechanisms with non-homologous end joining (NHEJ) (Lieber, 2010) or homology directed repair (HDR) (Moynahan and Jasin, 2010), enabling precise genome modification.

Distinct from the site-specific nucleases described above, the CRISPR-Cas system, an RNA-guided DNA endonuclease, is more efficient and simpler for designing system, that has been rapidly exploited as a genome engineering system in a short period of time and applied to many different kinds of living cells and organisms. Currently, CRISPR-Cas9 and CRISPR-Cpf1 systems from bacteria have been developed as efficient genome editing tools for a wide range of species in three domains of life (Jinek et al., 2013; Doudna and Charpentier, 2014; Shalem et al., 2014; Singh et al., 2017, 2018).

However, CRISPR-Cas9 or Cpf1 is not always effective in bacterial and archaeal cells, especially in extremophilic microorganisms. Therefore, an alternative strategy for genome editing in these prokaryotic cells is using their endogenous CRISPR-Cas systems. This strategy only requires shuttle vectors expressing the corresponding CRISPR RNA matching the target site and carrying the repairing donor DNA. The CRISPR RNA is processed by the endogenous ribonuclease. The first report of genome editing using an endogenous CRISPR-Cas system employed Cas9 in *Streptococcus pneumoniae* (Jiang et al., 2013). In the bacterium *Clostridium pasteurianum*, its endogenous subtype I-B CRISPR-Cas system resulted in superior genome editing efficiency compared to using the CRISPR-Cas9 system from *Streptococcus pyogenes* (Pyne et al., 2016). A self-targeted genome editing technology has been successfully established in *Streptococcus mutans* using its endogenous II-A CRISPR-Cas system (Gong et al., 2018). Otherwise, an exploitation of the type I-B CRISPR-Cas system for multiplex genome editing to engineer high-level butanol production in *Clostridium tyrobutyricum* has been reported (Zhang et al., 2018). Recently, the endogenous subtype I-E system was employed to edit the genetically recalcitrant species *Lactobacillus crispatus* with high efficiency (Hidalgo-Cantabrana et al., 2019).

Endogenous CRISPR-based genetic tools are especially suitable for extremophilic bacteria or archaea, because Cas9 or Cpf1 from mesophilic bacteria do not work well in these cells. Therefore, our group explored the endogenous type I and III systems in the thermophilic archaeon *Sulfolobus islandicus* for high-efficiency genome editing (Li et al., 2016). Similarly, the haloarchaeal I-B CRISPR-Cas system was harnessed for efficient genome editing in this polyploid archaeon (Cheng et al., 2017). Altogether, given the wide diversity of native CRISPR-Cas systems in bacteria and archaea, this approach can be readily adapted to other prokaryotes for genome editing (Hidalgo-Cantabrana et al., 2018). Endogenous CRISPR-based genome editing methods are also suitable for newly isolated industrial strains with robust production efficiency but fewer genetic tools. For example, low efficiency of genome editing was found in *Zymomonas mobilis* and *Pediococcus acidilactici*, important for ethanol or lactic acid fermentation, using CRISPR-Cas9 or double-crossover recombination methods. Using their endogenous subtype I-F or II-A systems, high-efficient genome editing has been achieved (Liu et al. and Wang et al., unpublished).

## CRISPR-Based Antimicrobials

Pathogens showing antimicrobial resistance are called superbugs. The development of antimicrobial resistant bacteria (AMRB) poses an increasingly serious threat to global public health (Friedman, 2015; Jans et al., 2018). Phage therapy against superbugs is an important method (Mertz, 2019); however, CRISPR-Cas systems can be repurposed to specifically target AMRB genomic DNA (or RNA) to combat antibiotic resistance and kill them (de la Fuente-Nunez and Lu, 2017). To develop CRISPR-based antimicrobials, successful delivery of self-targeting mini-CRISPR DNA is crucial. Development of non-viral delivery strategies and the engineering of bacteriophages present potential solutions. Several non-viral materials as vehicles of delivery to microorganisms have been reported, such as the use of polymer-derivatized Cas9 protein by direct covalent modification for treating multidrug-resistant bacteria (Kang et al., 2017), gold nanoparticles (Wang et al., 2018), and other lipid formulations. Another alternative is bacteriophages, which have been widely used for gene delivery and the treatment of pathogenic bacterial infections (Westwater et al., 2003; Lu and Collins, 2007). Especially, many AMRBs, including *Staphylococcus aureus*, *Enterococcus faecium*, *Klebsiella pneumonia*, and *Mycobacterium tuberculosis*, encode endogenous CRISPR-Cas systems^1^. Therefore, transduction of phages or transformation of plasmids expressing artificial crRNA matching superbugs’ conserved DNA sequence can kill AMRB *via* the endogenous CRISPR-Cas systems without requirement of an additional CRISPR nuclease. As an example, using the *E. coli* endogenous subtype I-E CRISPR-Cas system, individual bacterial strains and species with specific sequences in the genome were targeted and removed, no matter the genomic location, strand, or transcriptional activity of the target site (Gomaa et al., 2014). A M13-based phagemid was used to deliver the CRISPR-Cas system to target antibiotic resistance plasmids and chromosomal genes in pathogenic *E. coli*, leading to antibiotic sensitivity and death (Citorik et al., 2014). At the same time, similar work using heterogeneous CRISPR-Cas carried by *Staphylococcus aureus* φ NM1 phage-based phagemid to target the pathogenic host was reported (Bikard et al., 2014). In short, CRISPR-Cas systems, the pathogen’s defenses, were turned into a weapon against the pathogen itself (Greene, 2018).

**Figure 2:**
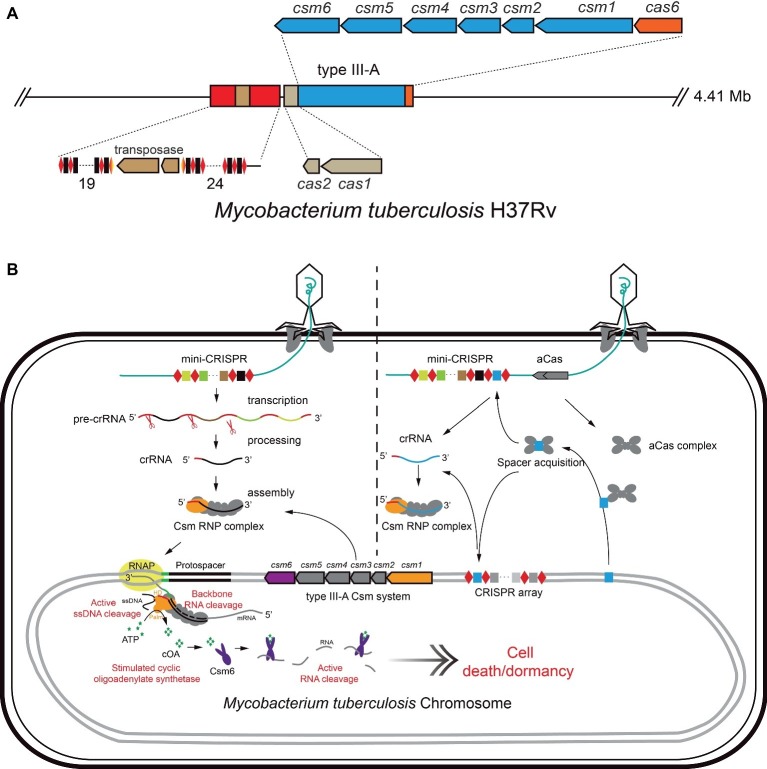
Schematic of endogenous CRISPR-Cas system-based method for elimination of *M. tuberculosis*. **(A)** The CRISPR locus of *M. tuberculosis* includes two CRISPR loci and *cas* genes encoding Cas1/Cas2 (Adaptation module), Csm1-5 (type III-A interference module), Csm6 (ancillary ribonuclease), and Cas6 (crRNA processing). **(B)** The mini-CRISPR against the essential DNA of *M. tuberculosis* was delivered by mycobacteriophage. After transduction, mini-CRISPR was replicated with phage DNA and transcribed. The CRISPR RNA was processed by the endogenous Cas nuclease (eg. Cas6). Then, the endogenous Csm complex binds mature crRNA to form Csm RNP complex (model on the left). Alternatively, expression of CRISPR-Cas adaptation proteins leads to spacer acquisition from the chromosome, facilitating the self-targeting activity of endogenous CRISPR-Cas systems in the cell (model on the right). Csm RNP recognizes and cleaves the target RNA, inducing cleavage of non-specific single-strand DNA in cells. This process will induce synthesis of cOAs by the Cas10 subunit of Csm RNP complex. cOAs activate the non-specific ribonuclease activity of Csm6, resulting in cell dormancy or death.

Tuberculosis (TB) is a serious infectious respiratory disease caused by *M. tuberculosis*, which has developed resistance to the most effective first-line antibiotic rifampicin, and has multidrug-resistance. Gene silencing was achieved by employing the heterogenous dCas9 system in mycobacteria for understanding the gene function (Choudhary et al., 2015, 2019) and tuberculosis drug discovery (Rock et al., 2017; McNeil and Cook, 2019; Rock, 2019). Here, we use an ongoing work in our lab as an example, to introduce the strategies and principles of an endogenous CRISPR-Cas system-based genetic tool as an antimicrobial against *Mycobacterium tuberculosis*. *M. tuberculosis* encodes a subtype III-A CRISPR-Cas system, including two CRISPR loci, six *csm* genes for target interference, one *cas6* gene for crRNA processing, and an adaptation module (Figure 2A; He et al., 2012; Gruschow et al., 2019; Wei et al., 2019). As mentioned above, the subtype III-A CRISPR-Cas system provides a strong defense against targets including RNA, non-specific ssDNA and RNA (Kazlauskiene et al., 2016; Liu et al., 2017c; Huo et al., 2018; Jia et al., 2018, 2019; Gruschow et al., 2019). Additionally, *M. tuberculosis* has a rich bacteriophage population, which is convenient for selecting a phage vector for delivery of gDNA against the host chromosome.

To achieve this purpose, the plasmid phAE159, derived from mycobacteriophage TM4 (PH101ts) (Bardarov et al., 2002), can be used as the delivery vector. An artificial mini-CRISPR array harboring the “Repeat-Spacer-Repeat” cassette including the spacer can target an essential gene (such as RNA polymerase gene, etc.) in the genome of *M. tuberculosis*. The recombinant phagemid can be transfected into *M. smegmatis* mc^2^155 at 30°C to obtain mycobacteriophage plaques for high-titer phage stock production (Jain et al., 2014). Then, the anti-tuberculosis bacteriophages can be delivered by different inhalation devices to compare titer reduction and delivery rate (Carrigy et al., 2017). Once the recombinant mycobacteriophage infect *M. tuberculosis*, crRNAs expressed from the artificial mini-CRISPR array will guide the endogenous CRISPR-Cas system to target the protospacer, and the multiple self-targeting activity of Csm-RNP complex would kill cells efficiently, as shown in Figure 2B. Furthermore, RNA cleavage mediated DNA interference will generate cyclic oligoadenylate as the messenger to activate the non-specific RNA interference activity of Csm ribonuclease, resulting in dormancy or death of *M. tuberculosis* cells.

## Final Remarks and Prospect

Within the past few years, CRISPR-Cas systems have formed a revolutionary genome editing tool, giving a great impetus to the development of life science and our understanding of life. Beyond the Class 2 CRISPR-Cas systems, which are widely used for genome editing with single protein RNP complexes, more active endogenous CRISPR-Cas system can readily be applied in genome editing in other bacteria and archaea, especially species with limited activity of heterogeneous Cas proteins (Selle and Barrangou, 2015; Li et al., 2016). CRISPR-Cas systems provided efficient interference in precise chromosomal sites, after repurposing them as antimicrobials (Yosef et al., 2015). There are some obstacles to the efficient application of this technology in other new species that need to be addressed. First, in order to make best use of the endogenous CRISPR-Cas systems for genome editing and antimicrobials, the specificity of PAM sequence and the activity of Cas proteins must be fully characterized that can be tested and predicted by plasmid interference assays or bioinformatics retrieval. Second, highly efficient delivery systems are required for delivering CRISPR-Cas modules (nuclease genes and mini-CRISPR, or only mini-CRISPR). Phage vectors are preferred since phages can widely propagate and infect resistant bacteria. In addition, escape from CRISPR-Cas interference through mutations and deletions in spacer or protospacer is always found (Gudbergsdottir et al., 2011; Semenova et al., 2011; Stout et al., 2018), leading to failure to kill resistant bacteria. Phage therapy in combination with CRISPR-Cas interference may be one solution. CRISPR-Cas systems also acquire new spacers from their host genome, resulting in self-targeting (Horvath et al., 2008), although they utilize multiple mechanisms to preferentially acquire spacers from foreign DNA, such as primed adaptation (Datsenko et al., 2012). Self-spacer adaptation was detected in the *Sulfolobus* subtype I-A system (Liu et al., 2015; Zhang et al., 2019) and *Streptococcus* type II-A system (Wei et al., 2015), when the adaptation system was activated. Therefore, activation of the endogenous or heterogeneous CRISPR adaptation module in the resistant bacteria will spontaneously uptake spacers from pathogenic bacteria (such as *M. tuberculosis*) genomic DNA into the CRISPR array, either on the genome or on the vector DNA, resulting in killing the pathogenic bacteria themselves (Figure 2B). Improving this technology is of great significance in solving the hazards of drug-resistant bacteria.

## Author Contributions

YL collected the literature and wrote the manuscript while NP reviewed and edited manuscript. YL and NP developed the ideas presented in this manuscript, read and approved the final manuscript.

### Conflict of Interest

The authors declare that the research was conducted in the absence of any commercial or financial relationships that could be construed as a potential conflict of interest.

## Footnotes

^1^https://crispr.i2bc.paris-saclay.fr/
